# Hypnotism in Dentistry

**Published:** 1890-08

**Authors:** 


ARTICLE VII.

### HYPNOTISM IN DENTISTRY.

DR. HUGENSCHMIDT, of Paris, writing in the *Dental Review* says ;—It is a well recognized fact, by all those who have carefully studied the subject of hypnotism, that certain persons can hypnotize *themselves*, without the aid or presence of a second person.

The hypnotic state being obtained, insensibility of a certain part of the body can be easily produced, just by the verbal statement of the operator who simply says: "This or that part has become insensible to pain." At this moment the operation can be performed painlessly.

In certain cases the patient, as soon as hypnotized, is in a general anæsthetic condition.

I have now studied this method for over seven years in general medical practice, but have only once employed it in dentistry, this being in a general hospital. I object to using this method in our office, on account of the general superstition still attached to this subject of hypnotism or magnetism by a majority of patients, who believe that one who occupies himself with hypnotism is endowed with a "supernatural power," rendering him capable of doing any thing with a patient who places herself or himself into his hands. It is needless to say that this is an erroneous idea; yet as in our specialty, we are most of the time alone in our office with a patient, I have preferred to keep on the safe side, and, to prevent any disagreeable allusion, I have always positively refused to employ hypnotic sleep in our office for a dental operation, although I am absolutely convinced that this method would be of the greatest value in our specialty, especially in painful or very long operations.

M. Godon, in a communication read before the Odontological Society of Paris, in May, 1888, (*Odontologie*, July 1888), speaks on the use of hypnotism in dental surgery, mentioning the fact that this method was then being used at the Paris Dental School for the painless extraction of teeth. M. Godon also reported a case in which he used, on several occasions, mental suggestion on the same person for the purpose of excavating sensitive dentine on a highly nervous patient with complete success.

The only time I used this method in our specialty, as stated above, was in a military hospital of Paris, in 1882, on a soldier who was suffering severely from an exposed pulp in a superior bicuspid.

Before hypnotizing the patient I slightly touched the pulp with a needle. The way the patient expressed his delight in regard to my proceeding proved to me that the pulp was by far not a dead one. The man was then hypnotized. When he was placed in that condition I was able to touch the pulp, to pinch it, to tear it. I introduced a needle in the middle of its substance; it bled, but not the slightest indication of sensation could be denoted on the patient's countenance. He was then again restored to consciousness—to his normal state—and the needle was again introduced into the pulp canal. A formidable cry, accompanied by a good, solid profane word, was highly expressive of the variation of sensibility of our patient's pulp.—*The Dental Record.*

